# Simultaneous determination of multiple marker constituents in concentrated *Gegen Tang *granule by high performance liquid chromatography

**DOI:** 10.1186/1749-8546-2-7

**Published:** 2007-06-20

**Authors:** Jingzheng Song, Quanbin Han, Chunfeng Qiao, Yuekeung Yip, Hongxi Xu

**Affiliations:** 1 Chinese Medicine Laboratory, Hong Kong Jockey Club Institute of Chinese Medicine, Shatin, NT, Hong Kong SAR, China

## Abstract

**Background:**

Concentrated *Gegen Tang *(*GT*) granule is a widely available traditional Chinese medicinal product for the treatment of cold and flu. There was no reliable analytical method available for the quality assessment of *GT *granules.

**Methods:**

An HPLC method with an Agilent Zorbax SB-Phenyl Stablebond column (250 × 4.6 mm, 5 μm) was developed and validated. The mobile phase gradient was a mixture of 0.1% trifluoroacetic acid (TFA) in acetonitrile (ACN) and 0.1% TFA in water. The detection with a diode-array detector was set at 207, 230, 250 and 275 nm. Seven components, namely puerarin, daidzein, paeoniflorin, cinnamic acid, glycyrrhizin, ephedrine and pseudoephedrine were selected as marker compounds for the evaluation.

**Results:**

The regression equations revealed good linear relationships (correlation coefficients: 0.9994–0.9998) between the peak areas and concentrations. The recovery was between 98.8% and 101.7% with good precision and accuracy. The quality of *GT *granule from four different manufacturers was evaluated with this newly developed method. Samples from four manufacturers showed similar profiles but contents of the detected markers varied significantly among manufacturers and batches.

**Conclusion:**

A new method using high performance liquid chromatography (HPLC) has been developed for simple and reliable quality control of commercial concentrated *GT *granules. Sensitivity was increased by multi-wavelength detection. The contents of selected marker components in *GT *granules varied significantly among manufacturers and batches, making it necessary to evaluate the quality of concentrated *GT *granules in the market.

## Background

**Figure 1:**
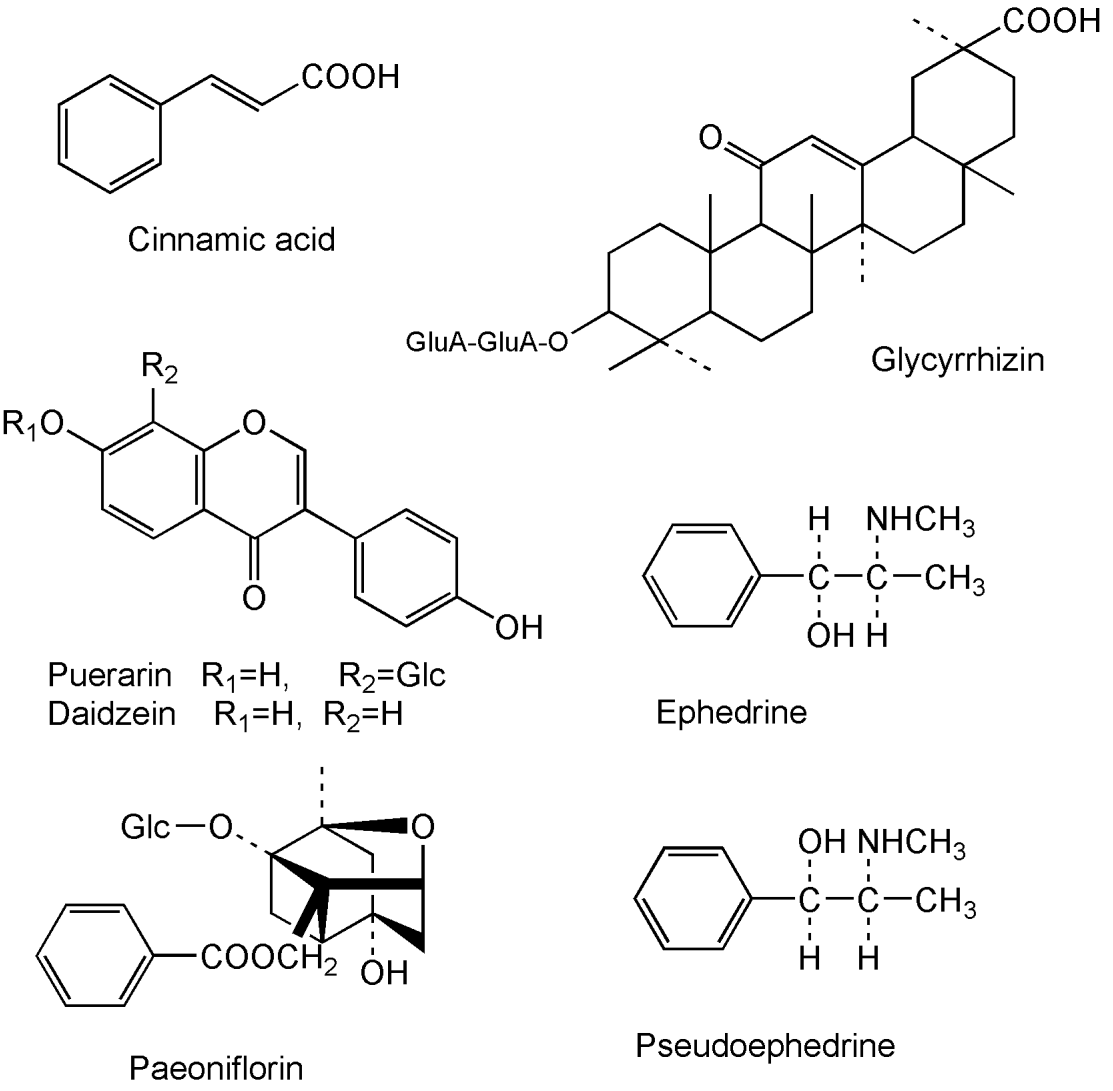
Chemical structures of seven chemical markers in *Gegen Tang *granule.

Listed in *Shanghan Lun *(*Discussion of Cold-induced Disorders*) by Zhang Zhongjing of the Han Dynasty, *Gegen Tang *(*GT*), a decoction of *Radix Pueraria Lobatae *(*Gegen*), is a classical formula of traditional Chinese medicine. It is widely used in China and South East Asia for treating cold and flu [1]. Modern pharmacological studies have revealed that it can alleviate upper respiratory tract diseases, trigeminal neuralgia, lumbar muscle strains, tension headaches, arthralgia, ischemic stroke, rheumatoid arthritis, dysentery and nettle rash. It also has antiviral, anti-pathogen, anti-inflammatory, and anticoagulant activities [2-9]. Some studies indicate that these effects are attributed to the major components in this preparation, such as puerarin, daidzein, paeoniflorin, cinnamic acid, glycyrrhizin, ephedrine and pseudoephedrine [2,10-12], the chemical structures of which are shown in Figure 1. In recent years, the formula of *GT *has been developed into a concentrated granule form which has advantage over the traditional decoction.

The formula of *GT *consists of seven herbs (Table 1). According to traditional Chinese Medicine theories, *Radix Puerariae *(*Gegen*) is the 'primary' herb in the formula, whereas *Herba Ephedrae *(*Mahuang*) and *Ramulus Cinnamomi Cassiae *(*Guizhi*) are 'deputies' to help release the 'primary' herb and relieve the muscle layer. *Radix Paeoniae Alba *(*Baishao*), *Rhizoma Zingiberis Recens *(*Shengjiang*) and *Fructus Zizyphi Jujubae *(*Dazao*) are 'assistant' herbs while *Radix Glycyrrhizae *(*Gancao*) is the 'envoy' [1].

**Table 1:** Herbal composition and daily dosage of *Gegen Tang *granule from four manufacturers

**Ingredients**	A*	**B**	**C**	**D**
*Radix Puerarae *(*Gegen*)	-	22.7%	32.0%	21.4%
*Herba Ephedrae *(*Mahuang*)	-	16.6%	16.0%	16.1%
*Radix Glycyrrhizae *(*Gancao*)	-	10.7%	8.0%	10.7%
*Radix Paeoniae Alba *(*Baishao*)	-	11.0%	12.0%	10.7%
*Ramulus Cinnamomi Cassiae *(*Guizhi*)	-	11.0%	12.0%	10.7%
*Rhizoma Zingiberis Recens *(*Shengjiang*)	-	16.6%	4.0%	16.1%
*Frutus Jujubae *(*Dazao*)	-	11.0%	16.0%	14.3%
Daily dosage	2 × 4 g	2 × 5 g	3 × 3 g	2 × 5 g

The percentage of each herbal ingredient was collected from the insert (B and C), or calculated after normalized total to 100% (D).

*The composition of A is unknown.

The quality of the raw herbs is one of the major factors for the quality of proprietary preparations of traditional Chinese medicines. For example, roots from *Puerariae lobata *(Willd.) Ohwi and *P. thomsonii *Benth are used as the source of *Radix Puerariae*. However, the contents of puerarin and daidzein, two major marker components in *Radix Puerariae Lobatae*, were 8-fold and 5-fold higher than those in *Radix Puerariae Thomsonii *(*Fenge*) respectively [13]. An interesting study revealed that *Radix Puerariae Lobatae *was much more effective in relieving fever than *Radix Puerariae Thomsonii *[13]. Furthermore, the regions where the raw materials were collected and the season when they were collected may also affect the quality of the preparations. As few analytical methods are available for the quality control of *GT *granules, the quality of concentrated *GT *granules in the market still remain questionable.

As a popular tool for the quality control of traditional Chinese medicines, high performance liquid chromatography (HPLC) was used for the determination of *GT*. However, its procedure was very complicated [14,15]. A micellar electrokinetic chromatography (MEKC) method was also used for the determination of seven marker compounds in *GT *granules [16]. Between the two methods, the precision and accuracy of the MEKC method were not satisfactory for quantitative analysis and its sensitivity was inferior to that of HPLC.

As concentrated *GT *granule is popularly used as a non-prescriptive drug in China and East Asia, its quality control is vitally important. In this paper, we report an HPLC method first developed and validated for the quality control of *GT *granule. While often used as specific marker compounds for conventional quality control of the respective herbs as well as proprietary Chinese medicinal preparations, seven components were simultaneously determined in this new method. Extraction efficiency of these markers was optimized through comparisons of extraction of granules with different solvents and time intervals. Finally, the quality of *GT *granules was evaluated under the optimized HPLC method.

## Methods

### Material and reagents

Fourteen batches of concentrated *GT *granules manufactured by four different manufacturers (code named A, B, C and D) were purchased from local pharmacies in Hong Kong. The reference standards of puerarin, daidzein, paeoniflorin, cinnamic acid and glycyrrhizin, were purchased from the National Institute for the Control of Pharmaceutical and Biological Products of China (Beijing, China). Ephedrine and pseudoephedrine were purchased from Sigma Chemical Co. (St. Louis, USA). The purity for all reference standards was over 98%. Trifluroacetic acid (HPLC grade) was purchased from International Laboratory (San Bruno, USA). HPLC-grade methanol and acetonitrile were obtained from Duksan chemical Co. (Kyungki-Do, Korea). HPLC-grade water used throughout the study was obtained from a Milli-Q Reagent Water System (Millopore, USA).

### Instruments and chromatographic conditions

All separations were performed on a Waters 2695 Separations Module HPLC system equipped with a quaternary pump, autosampler, column temperature controller, an online degasser and a Waters 2996 Photodiode Array Detector (PDA). The analytical column was an Agilent Zorbax SB-Phenyl Stablebond column (250 × 4.6 mm, 5 μm).

The mobile phase was composed of (A) water, (B) 0.1% TFA in water and (C) 0.1% TFA in ACN (A-B-C: 0 min 75:20:5; 25 min 60:20:20; 50 min 17:20:63; v/v/v). The flow rate was 1.0 mL/min. The column effluents were simultaneously monitored at 207 nm (for ephedrine and pseudoephedrine), 230 nm (for paeoniflorin), 275 nm (for cinnamic acid) and 250 nm (for other components). Content of each component was calculated on the basis of the absorbance at each respective selected wavelength. Injection volume was 10 μL. The column temperature was controlled at 25°C.

### Preparation of standard solutions

The standard stock solutions (1 mg/mL) were prepared by dissolving 5 mg of each reference standard to 5 mL of methanol. The stock solutions were stored at 4°C and remained stable for at least one month. The working standard solutions containing each of the seven reference standards were prepared by diluting the standard stock solutions to give serial concentrations within ranges listed in Table 2. The standard curve was analyzed with Excel using the linear least-squares regression equation derived from the peak areas.

**Table 2:** Regression equation, correlation coefficients, linearity ranges and limit of detection and quantitation for the markers of *Gegen Tang *granule

**Compounds**	**Regression equation**	**Linear range (μg/mL)**	**R^2^**	**LOD (ng/mL)**	**LOQ (ng/mL)**	**Intra-day RSD% (n = 6)**	**Inter-day RSD% (n = 3)**
Puerarin	*y *= 40149*x *– 5483	1.70 – 69.70	0.9997	6.21	20.70	0.83	0.78
Paeoniflorin	*y *= 13018*x *+ 3168	1.07 – 64.36	0.9996	19.64	65.46	0.99	0.45
Cinnamic acid	*y *= 68413*x *+ 2472	0.15 – 5.81	0.9998	3.72	12.40	0.61	0.60
Daidzain	*y *= 44698*x *+ 350	0.06 – 4.96	0.9997	5.58	18.60	0.33	1.83
Glycyrrhizin	*y *= 5695*x *– 3089	1.07 – 85.26	0.9998	45.47	151.56	0.10	1.70
Ephedrine	*y *= 21411*x *+1949	1.08 – 86.13	0.9998	3.82	12.73	0.52	1.55
Pseudoephedrine	*y *= 30269*x *– 41321	2.98 – 119.13	0.9996	2.71	9.03	0.37	0.41

All regression results are statistically significant (P < 0.05)

Recovery of the seven marker components from *GT *granule was determined by spiking known amount of reference standards at three different concentrations (Table 3) to 20 mg of *GT *granule with known contents of the seven markers.

**Table 3:** Recovery of seven markers in *Gegen Tang *sample*

**Compounds**	**Contents (μg/g)**	**RSD% (n = 6)**	**Added (μg/mL)**	**Obtained (μg/mL)**	**Recovery (± RSD %, n = 3)**
Puerarin	3920	0.61	3.50	3.54	101.20 ± 0.74
			8.75	8.68	99.26 ± 0.43
			13.85	13.75	99.41 ± 0.51
Paeoniflorin	3219	0.54	3.63	3.60	99.28 ± 1.61
			9.07	8.99	99.14 ± 1.07
			13.27	13.16	99.33 ± 0.51
Cinnamic acid	317	0.73	0.27	0.27	99.78 ± 1.01
			0.68	0.68	100.23 ± 1.67
			0.87	0.87	100.01 ± 1.06
Daidzain	283	1.14	0.19	0.19	100.35 ± 1.10
			0.48	0.48	98.94 ± 1.50
			0.78	0.77	98.76 ± 0.76
Glycyrrhizin	4138	0.55	3.56	3.59	100.92 ± 1.16
			8.89	8.93	100.54 ± 0.40
			16.96	17.09	100.73 ± 0.30
Ephedrine	4283	0.67	3.70	3.74	101.03 ± 0.83
			9.26	9.30	100.38 ± 1.34
			14.41	14.29	99.22 ± 1.35
Pseudoephedrine	1007	1.21	0.50	0.96	101.70 ± 1.17
			2.37	2.39	101.02 ± 2.01
			4.19	4.23	100.71 ± 0.72

*Sample Batch No. 4 from manufacturer D is used in this experiment.

### Sample preparation

Ten grams of each *GT *granule sample was ground to fine powder. Forty milligrams of the fine powder was accurately weighed and dissolved in 10 mL of 60% methanol (v/v) by ultrasonication for 30 min. After being cooled down to room temperature, the sample solution was centrifuged at 13000 rpm for 5 min and the supernatant was filtered through a 0.2 μm syringe membrane filter. Ten microliters of the filtrate was injected into the HPLC system for analysis.

Statistical analyses were performed using Data Analysis Toolpak Microsoft Excel for Windows. It is considered to be statistical significant when P value is less than 0.05.

## Results

### Optimization of chromatographic conditions

The mobile phase was optimized through comparisons of different solvents, solvent ratio and gradient profile. Compared with other solvents, acetonitrile showed the best separation, shortest analyzing time and lowest column pressure. An acidified mobile phase could minimize peak tailing and improve resolution. A simple gradient was used for single run elution within 50 min. A mixture of water, 0.1% TFA in water and 0.1% TFA in ACN were chosen as the mobile phase gradient. The resolution between cinnamic acid (peak 3) and daidzein (peak 4) was 1.3 and the resolutions between other peaks were larger than 2.0.

### Method Validation

#### Calibration

All calibration graphs were plotted based on linear regression analysis of the integrated peak areas (*y*) versus concentrations (*x*, μg/mL) of the seven markers in the standard solution at ten different concentrations. Results showed a good linear relationship between the peak area and concentration (Table 3).

The limit of detection (LOD) and limit of quantitation (LOQ) were determined with standard solution on the basis of a signal-to-noise ratio of 3 and 10 respectively. The values of LOD and LOQ were much lower than the reported methods [13-15], indicating that the proposed method was sensitive for the determination.

#### Precision

The intra-day precision was evaluated by determining a standard mixture solution of the seven markers under the optimized condition six times within a day. For inter-day precision, the measurement was conducted two times per day for three consecutive days. As shown in Table 2, the intra-day and inter-day relative standard deviations (RSDs) were 0.10–0.99% and 0.41–1.83% respectively.

#### Repeatability

Six independently prepared sample solutions of concentrated *GT *granules with the same amount were analyzed and the variations within six measurements were calculated for repeatability. The measurements followed those described in sample preparation. RSDs (parameters for repeatability) ranged from 0.54 to 1.21% (Table 3), indicating that the conditions used in the quantitative analysis were satisfactory.

#### Recovery

Recovery studies were carried out by spiking three different concentrations of the mixed standards to the *GT *granules sample. The average recoveries were 99.3–101.2% for puerarin, 99.1–99.3% for paeoniflorin, 99.8–100.2% for cinnamic acid, 98.8–100.4% for daidzein, 100.5–100.9% for glycyrrhizin, 99.2–100.4% for ephedrine and 100.1–101.7% for pseudoephedrine (Table 3). The results showed that the proposed method was accurate for the determination.

### Sample analysis

The extraction efficiency was evaluated by 100% methanol, 60% methanol and water. As regards the samples from manufacturers B, C or D, it was shown that the extraction efficiencies were similar among these three solvents and no obvious difference was observed among them. However, in the sample from manufacturer A, the extraction efficiency achieved by 60% methanol was much higher than those by other solvents. Thus, 60% methanol was chosen for extraction in this study.

Solvent volume and sonication time were also optimized (5, 10 and 20 mL of 60% methanol sonicated at 10, 20, 30 and 40 min). For the samples from manufacturers B, C and D, 20 min sonication produced the best yield. For the sample from manufacturer A, 30 min sonication was needed. As regards volume of solvent, 10 mL produced the best yield, sensitivity and precision.

**Figure 2:**
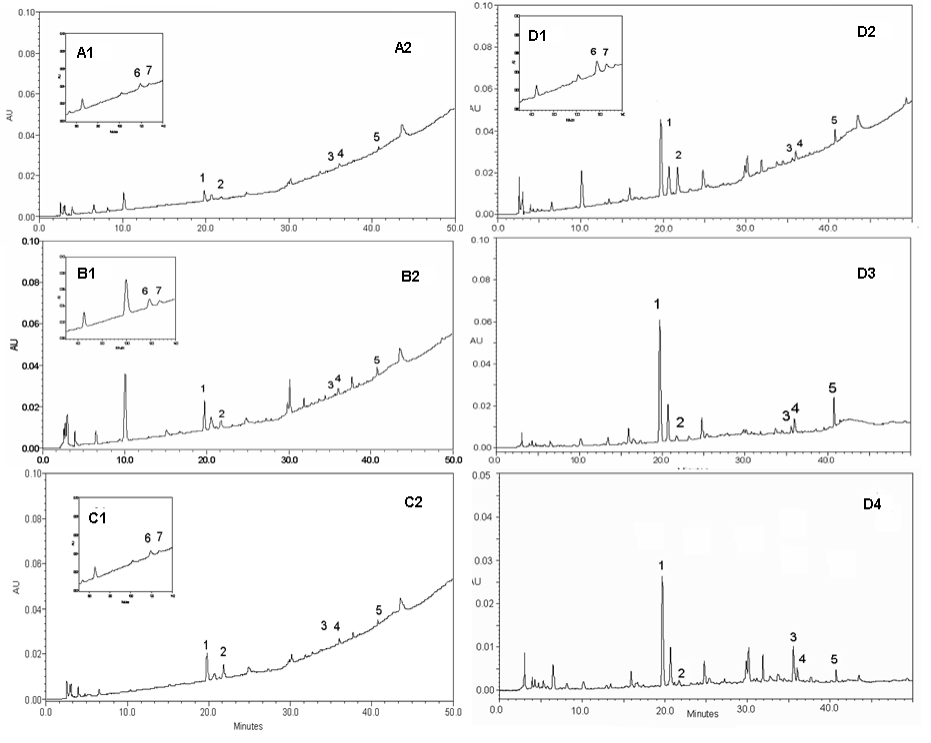
**HPLC chromatograms of seven marker compounds detected in different samples under the developed mobile phase system at different detection wavelengths**. Letters A, B, C and D denote the four manufacturers. Peaks: (1) Puerarin; (2) Paeoniflorin; (3) Cinnamic acid; (4) Daidzein; (5) Glycyrrhizin; (6) Ephedrine; (7) Pseudoephedrine. A2, B2, C2 and D2 are chromatograms monitored at 230 nm for the quantitative determination of paeoniflorin (peak 2) and also used for the comparison of different samples. A1, B1, C1 and D1 are chromatograms monitored at 207 nm for the determination of ephedrine (peak 6) and pseudoephedrine (peak 7). D3 is a chromatogram monitored at 250 nm for puerarin (peak 1), daidzein (peak 4) and glycyrrhizin (peak 5); and D4 is a chromatogram monitored at 275 nm for cinnamic acid (peak 3).

**Figure 3:**
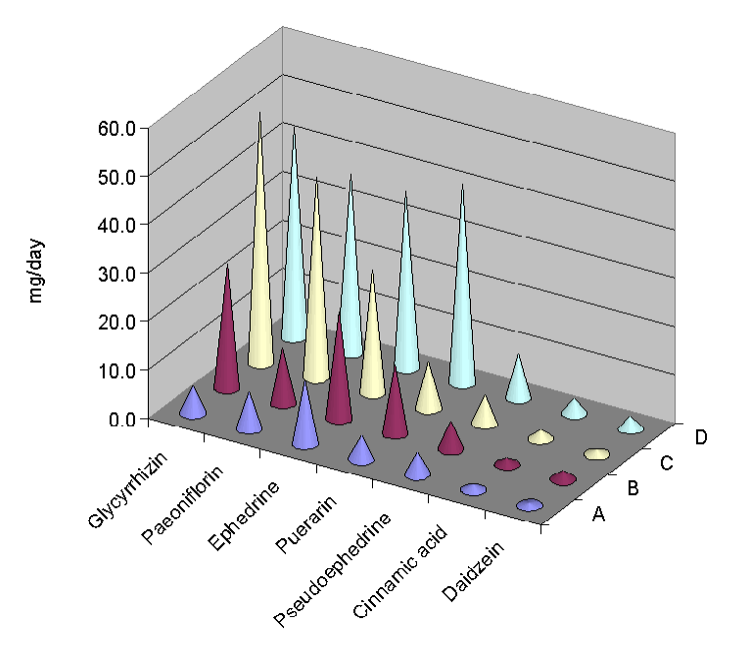
**Comparison of daily intake amounts of the seven compounds in *GT *granules from four manufacturers**. Letters A, B, C and D denote the four manufacturers. Values are means ± SD of batches from each manufacturer. Single factor ANOVA was performed on each compound. Contents of each compound showed significant differences among the four manufacturers (P < 0.05).

The quality of commercially available concentrated *GT *granules from the four manufacturers was evaluated by the established method. Typical chromatograms are shown in Figure 2. Although different samples from the four manufacturers showed a similar chromatographic profile, the peak areas were significantly varied. The contents of seven markers in different samples from different manufacturers were all within the ranges of the linear regression and much higher than LOQs (Table 4). In all samples, the major components were glycyrrhizin, puerarin, paeoniflorin and ephedrine. However, the contents of the measured constituents varied among batches within the *GT *granule from the same manufacturer. For example, the RSD for the samples from manufacturer D was less than 20%, which is an acceptable variation for a botanical product, whereas the RSDs for the samples from manufacturer A for the seven markers were 14.0–74.9% (Table 4). The average content of puerarin and glycyrrhizin in samples from manufacturer D, which showed the highest amount of markers, were six-fold higher than those from manufacturer A. In addition, daily intake amounts according to the daily recommended dosage (Table 1) were compared (Figure 3). The total daily intake amounts of the seven marker components in the samples from manufacturers A, B, C and D were 38.2, 83.2, 137.9 and 173.8 mg respectively.

**Table 4:** Contents (μg/g, mean ± SD, n = 3) of markers in *Gegen Tang *granule from four manufacturers

**Manufacturers and Batch No.**		**Puerarin**	**Paeoniflorin**	**Cinnamic acid**	**Daidzain**	**Glycyrrhizin**	**Ephedrine**	**Pseudoephedrine**
A*	Batch No.1	374.3 ± 3.1	270.5 ± 0.6	70.4 ± 0.4	52.1 ± 0.4	411.4 ± 2.8	731.4 ± 2.3	242.8 ± 2.3
	Batch No.2	440.8 ± 1.5	342.1 ± 2.7	89.7 ± 0.5	59.8 ± 0.5	542.6 ± 2.6	938.5 ± 8.8	322.4 ± 3.1
	Batch No.3	738.7 ± 4.1	353.8 ± 3.1	100.2 ± 0.7	131.4 ± 0.9	1558.3 ± 12.6	1162.0 ± 8.1	318.6 ± 2.0
	Mean (± SD)	517.9 ± 194.1	322.1 ± 45.1	86.8 ± 15.1	81.1 ± 43.7	837.4 ± 627.7	944.0 ± 215.4	294.6 ± 44.9
B*	Batch No.1	733.4 ± 5.8	423.1 ± 2.0	72.1 ± 3.8	63.2 ± 1.0	1584.3 ± 12.2	2121.0 ± 7.4	482.6 ± 4.2
	Batch No.2	2273.4 ± 21.4	1339.6 ± 4.2	183.4 ± 1.7	302.6 ± 0.7	3314.6 ± 14.6	2863.4 ± 21.8	891.4 ± 3.7
	Batch No.3	1708.6 ± 11.6	1030.7 ± 6.5	149.7 ± 0.9	228.7 ± 0.8	3276.5 ± 23.9	1822.6 ± 0.7	609.7 ± 3.1
	Mean (± SD)	1571.8 ± 779.1	931.1 ± 466.3	135.1 ± 57.1	198.2 ± 122.6	2725.1 ± 988.2	2269.0 ± 536.0	661.2 ± 209.2
C*	Batch No.1	1202.8 ± 14.0	4587.7 ± 16.1	224.1 ± 0.57	138.3 ± 0.8	5917.4 ± 39.1	2886.8 ± 0.7	743.7 ± 0.8
	Batch No.2	1479.4 ± 11.5	1672.4 ± 10.4	149.6 ± 0.7	162.6 ± 0.1	1814.2 ± 5.4	2293.7 ± 0.2	639.6 ± 4.5
	Mean (± SD)	1341.1 ± 19.6	3130.1 ± 2061.4	186.9 ± 52.7	151.5 ± 18.6	3865.8 ± 2901.4	2590.3 ± 419.4	691.7 ± 10.7
D**	Batch No.1	5913.6 ± 48.5	4899.4 ± 25.5	323.6 ± 1.9	324.1 ± 0.6	5087.1 ± 45.3	3740.4 ± 1.0	902.4 ± 8.1
	Batch No.2	3417.7 ± 18.5	4341.6 ± 24.7	238.9 ± 0.6	179.6 ± 0.3	4958.3 ± 15.4	3172.6 ± 0.4	1002.4 ± 6.1
	Batch No.3	3711.5 ± 18.6	4124.8 ± 32.5	271.4 ± 2.6	242.4 ± 0.9	4079.6 ± 11.8	4577.8 ± 0.8	1203.8 ± 6.3
	Batch No.4	3889.3 ± 19.4	3128.7 ± 21.0	287.6 ± 2.0	263.7 ± 0.8	4189.3 ± 20.5	4329.6 ± 0.4	969.7 ± 5.2
	Batch No.5	4193.2 ± 17.2	3623.3 ± 26.1	304.4 ± 2.7	269.9 ± 0.7	3958.7 ± 17.0	3820.3 ± 0.4	1134.3 ± 8.5
	Batch No.6	4362.7 ± 18.6	3411.7 ± 14.0	313.2 ± 2.3	324.1 ± 0.5	4672.7 ± 20.1	2617.8 ± 0.8	933.1 ± 4.9
	Batch No.7	4438.6 ± 31.1	3462.6 ± 7.6	327.8 ± 2.3	317.8 ± 0.6	4729.7 ± 8.5	2624.0 ± 0.8	959.7 ± 8.0
	Mean (± SD)	4275.2 ± 809.1	3856.0 ± 623.4	295.3 ± 31.8	274.5 ± 53.2	4525.0 ± 447.1	35554.6 ± 779.1	1015.1 ± 111.5

Values are means ± SD of triplicate tests. Single factor ANOVA was performed on each component. Contents of each compound showed significant differences among the four manufacturers (P < 0.05). * Significant differences were observed in different sample batches (P < 0.05). ** No significant difference was observed in the contents of each component in seven batches of *GT *from manufacturer D (P > 0.05).

## Discussion

Previously, two HPLC systems were applied for the determination of components in the decoction of *GT *using a C_18 _column with an ion-pair mobile phase to analyze ephedrine alkaloids [14], and a gradient mobile phase to separate non-alkaloids components [15]. These methods are time consuming and not suitable for *GT *granules. A MEKC method has been used for the determination of seven components in *GT *granules. Although the analysis time for the MEKC method was short, the reported recovery, precision and sensitivity were low. In our study, different types of columns, such as C_18 _and C_8_, were compared. The phenyl column showed a better simultaneous separation of all seven chemical markers.

Apart from puerarin and daidzein, the chemical structures of the markers are different (Figure 1). As the maximum absorbance wavelengths of these components are different, it is difficult to obtain a sensitive determination for all components with a single wavelength. Therefore, multi-wavelength detection was selected in order to obtain a sensitive determination for all components. The validated results showed that the proposed HPLC method was sensitive, precise, accurate and reliable for the quality assessment of *GT *granules.

The herbal compositions of the *GT *granules from the four manufacturers are similar (Table 1); however, the statistical analysis indicated that the contents of seven marker compounds (Table 4) and daily intake amounts of the marker compounds (Figure 3) were significantly different among the four manufacturers (P < 0.05). Moreover, significant differences were observed in different sample batches from manufacturers A, B and C (P < 0.05) respectively. Full assessment of the quality of *GT *granule preparations is therefore warranted. On the other hand, no significant differences (P > 0.05) of the contents of the marker compounds were found in samples of different batches from manufacturer D, an indication of better quality consistency from this manufacturer.

## Conclusion

A multi-component HPLC method with a simple extraction procedure has been developed for the simultaneous quantitation of seven markers in commercially available concentrated *GT *granules. Results indicate that the present method was precise, accurate and reliable in controlling the quality of *GT *granule. The quantitative results show that the contents of the seven markers in *GT *granules were significantly varied among the four manufacturers, as well as among batches from the same manufacturer. We conclude that this HPLC method is a reliable and simple method for the quality evaluation of concentrated *GT *granules.

## Competing interests

The author(s) declare that they have no competing interests.

## Authors' contributions

JS and YY performed sample collection, method development, validation, sample analysis and manuscript drafting. CQ and QH participated in the design of the study, chemical markers collection and manuscript drafting. HX conceived the idea of the study and participated in its design and coordination. All authors approved the final manuscript.
